# Varicella in Adult Foreigners at a Referral Hospital, Central Tokyo, Japan, 2012–2016

**DOI:** 10.3201/eid2601.170565

**Published:** 2020-01

**Authors:** Saho Takaya, Satoshi Kutsuna, Yuichi Katanami, Kei Yamamoto, Nozomi Takeshita, Kayoko Hayakawa, Yasuyuki Kato, Shuzo Kanagawa, Norio Ohmagari

**Affiliations:** National Center for Global Health and Medicine, Tokyo, Japan

**Keywords:** varicella, varicella zoster virus, VZV, viruses, vaccines, adult foreigners, emigrants and immigrants, students, epidemiology, Japan

## Abstract

We report a case series of varicella among adult foreigners at a referral hospital in central Tokyo, Japan, during 2012–2016. This series highlights differences in varicella vaccination schedules by country and epidemiology by climate and identifies immigrants and international students as high-risk populations for varicella.

Varicella is a benign disease with fever and rash caused by primary infection with varicella zoster virus (VZV) (*1*). Varicella is usually self-limiting but can sometimes be life-threatening, especially in adults (*1*). The World Health Organization estimates that 140 million varicella cases and 4,200 related deaths occur each year (*1*). In Japan, 1 million persons, primarily young children, have varicella every year. Of these patients, 4,000 require hospitalization, and adults make up ≈60% of this population (*2*).

Varicella vaccine is a live attenuated vaccine and is effective against varicella in healthy children (*1*). One dose of varicella vaccine reduces the number of varicella cases but can also reduce opportunities for exposure to the virus in the community and result in breakthrough varicella. Therefore, many industrialized countries now recommend 2 doses of varicella vaccine (*2*). In Japan, the 2-dose varicella vaccination became part of the childhood immunization schedule in October 2014; the number of varicella cases has drastically decreased since implementation (*3*).

We report cases of varicella in adult foreigners who were given a diagnosis at the National Center for Global Health and Medicine (NCGM) Medical Center in central Tokyo, Japan. These cases highlight major health issues, including differences in varicella vaccination programs in countries and VZV epidemiology in temperate and tropical regions, and identify immigrants and international students as high-risk populations for varicella.

## The Study

We reviewed 22 varicella cases in adult foreigners diagnosed during January 2012–December 2016 at the NCGM. During the same period, 7 cases of varicella were diagnosed in Japanese adults at the NCGM. We compiled basic demographic information for the patients (Table). Eleven case-patients were from Vietnam, 5 from China, and 1 each from 6 other countries (Cambodia, New Zealand, Belgium, Italy, Uzbekistan, and Nepal). Among 21 patients who resided in central Tokyo, 18 were international students. Fourteen patients were male, and the median age was 19 years (range 18–35 years).

**Table:** Characteristics for 22 case-patients (adult foreigners) with varicella, National Center for Global Health and Medicine, Tokyo, Japan, 2012–2016*

Case-patient no.	Time of onset	Days after onset of index case	Age, y/sex	Country of origin	Status or occupation	Exposure source	History of varicella	Previous varicella vaccination	Hospitalization (reason)
1	2012 Apr	Not applicable	19/M	Cambodia	Student	Sibling	NA	NA	Yes (infection control)
2	2012 Jul	Not applicable	32/F	China	Housewife	Own child	No	NA	No
3	2014 Apr	Index case	18/M	Vietnam	Student	UNK	No	UNK	No
4	2014 Apr	15	18/M	Vietnam	Student	School dormitory	No	UNK	No
5	2014 Apr	15	18/M	Vietnam	Student	School dormitory	NA	NA	No
6	2014 May	19	18/M	Vietnam	Student	School dormitory	NA	NA	No
7	2014 May	35	19/M	Vietnam	Student	School	NA	NA	No
8	2014 May	41	19/F	Vietnam	Student	School dormitory	NA	UNK	No
9	2014 May	42	18/F	Vietnam	Student	School dormitory	NA	UNK	No
10	2014 Jun	52	19/M	Vietnam	Student	School	NA	NA	No
11	2014 Jun	55	23/M	Vietnam	Student	School dormitory	NA	NA	No
12	2014 Jun	56	19/M	China	Student	School dormitory	NA	NA	No
13	2016 Mar	Not applicable	18/F	Vietnam	Student	UNK	No	No	No
14	2016 Mar	Not applicable	19/F	New Zealand	Student	UNK	No	No	Yes (malaise)
15	2016 Apr	Index case	35/F	China	Housewife	UNK	No	No	Yes (malaise)
16	2016 Apr	12	34/M	China	Unemployed	Patient 15 (wife)	No	Yes	No
17	2016 May	Not applicable	19/M	China	Student	UNK	No	No	Yes (malaise, headache)
18	2016 May	Not applicable	33/M	Belgium	Tourist	UNK	UNK	NA	No
19	2016 Jul	Not applicable	21/F	Italy	Student	UNK	No	No	Yes (infection control)
20	2016 Aug	Not applicable	20/M	Uzbekistan	Student	UNK	Yes	No	No
21	2016 Sep	Not applicable	23/M	Vietnam	Student	UNK	No	UNK	No
22	2016 Oct	Not applicable	25/F	Nepal	Sales clerk	UNK	No	No	No

*Case-patients 3–12 were from an outbreak in the same language school. Not applicable indicates sporadic cases, not cluster cases. NA, not available; UNK, unknown.

The patients sought medical care on median day 2 of illness (range days 1–5). Most diagnoses of varicella were made clinically; for some case-patients, varicella was confirmed by serologic testing or PCR or antigen detection testing of skin lesions. Twenty patients received antiviral therapy (acyclovir or valacyclovir). Five case-patients were hospitalized because of severe malaise and for infection control to prevent secondary cases.

Nine patients from Vietnam and 1 from China (case-patients 3–12) were students at the same language school who lived together in the school dormitories. During this outbreak, varicella developed in 9 students during a 2-month period after the index case (case 3). Information about previous vaccination and past infection could not be obtained for most patients in this outbreak because of their inability to communicate in Japanese or English and our inability to organize translation in the first language of the patients immediately at their visits. Overall, we found that there were 81 international students in 3 dormitories: 30 (37.0%) reported previous varicella and 6 (7.4%) claimed to have been vaccinated at least once. Although we contacted the school to provide postexposure prophylaxis, none of 41 students without previous VZV infection had received it.

## Conclusions

Japan has >2 million foreign residents; most are from China (30%), South Korea (20%), and the Philippines (10%) (*4*). However, Japan does not have any vaccination requirements for foreign residents. Because varicella is not a target of the World Health Organization Expanded Program on Immunization, the vaccine is not included in the national immunization schedules of many countries.

In China, from which Japan receives most of its immigrants and tourists, varicella vaccine was approved in the late 1990s and has been given on a voluntary basis (*5*). Although 80% of children in kindergartens and primary schools in Beijing were reported to have received the varicella vaccine, most had only received 1 dose, and breakthrough varicella has become a public health concern (*5*). In South Korea, the single-dose varicella vaccination was included in the national immunization program in 2005 and is recommended for 12–15-month-old children (*6*). Despite high use of the vaccine (>95%), varicella outbreaks continuously occur (*6*). The varicella vaccine has not been introduced into the national vaccination program of the Philippines (*7*).

The epidemiology of varicella differs between temperate and tropical regions. In temperate areas, most of the population contracts VZV before adolescence. In tropical areas, childhood varicella is much less common, and many persons stay susceptible to VZV until adulthood (*1*,*8*). This difference is attributed to changes in VZV transmissibility by temperature and humidity, population density, and sociologic factors, such as nursery school attendance (*8*).

Previous research has shown immigrants and refugees to be at high risk for varicella. A study in Denmark during the 1980s investigated an epidemic of varicella among refugees during a preasylum phase (*9*). The study showed that 44% of Tamil refugees from Sri Lanka had varicella infections within 1 week to 5 months after arrival in Denmark. Considering that many immigrants contracted varicella shortly after their arrival, it was deemed necessary to check VZV immunity for immigrants before they entered that country, rather than after entry.

In an outbreak study of 18 varicella cases among Mexican-born workers in Alabama, USA (*10*), Mexican-born workers were 5 times more likely to be susceptible to VZV than US-born workers. Although postexposure prophylaxis vaccine was provided to susceptible workers, a second dose was not administered to many of those who needed it. This finding shows the difficulty in reaching these populations because of their lack of stable access to healthcare. Faced with a rapidly aging population, Japan has also been accepting nurses and caregivers from countries in Southeast Asia as medical personnel in healthcare facilities and nursing homes (*11*). Thus, nosocomial infection with VZV is also a matter of concern.

Japan had 208,000 international students during 2015 (*12*). More than 90% of these students were from countries in Asia; most were from China (45%), Vietnam (19%), and Nepal (8%). The demographics of the case-patients in our study were consistent with these data. In general, colleges are suitable environments for communicable disease outbreaks because of highly dense interactions among students and staff, crowded living conditions, and an influx of students from many countries (*13*). Because 10%–20% of persons in Japan 15–30 years of age are susceptible to VZV (*14*), varicella outbreaks among international students might spread into the local student population. Despite these facts, Japan does not have any regulations or recommendations regarding the health of international students. In the United States, the American College Health Association strongly recommends institutional prematriculation immunizations (*15*). These guidelines recommend that all college students should have 2 doses of varicella vaccine unless they have other evidence of VZV immunity.

This varicella case series occurred in multifactorial contexts. However, little is known about the immunity against VZV among foreign residents in Japan. Educational institutions that receive international students need to consider varicella as a major preventable health issue among their students. Healthcare providers for immigrants and international students should also be aware of the risk for varicella and should verify VZV immunity or varicella vaccination status for students.
